# Ribosomal proteins in hepatocellular carcinoma: mysterious but promising

**DOI:** 10.1186/s13578-024-01316-3

**Published:** 2024-11-01

**Authors:** Qian Su, Huizhen Sun, Ling Mei, Ying Yan, Huimin Ji, Le Chang, Lunan Wang

**Affiliations:** 1 National Center for Clinical Laboratories, Institute of Geriatric Medicine, Chinese Academy of Medical Sciences, Beijing Hospital/ National Center of Gerontology, Beijing, P.R. China; 2 Beijing Engineering Research Center of Laboratory Medicine, Beijing Hospital, Beijing, P.R. China; 3 National Center for Clinical Laboratories, Peking Union Medical College, Chinese Academy of Medical Sciences, Beijing, P.R. China

**Keywords:** Hepatocellular carcinoma, Ribosomal protein, Biomarkers

## Abstract

Ribosomal proteins (RPs) are essential components of ribosomes, playing a role not only in ribosome biosynthesis, but also in various extra-ribosomal functions, some of which are implicated in the development of different types of tumors. As universally acknowledged, hepatocellular carcinoma (HCC) has been garnering global attention due to its complex pathogenesis and challenging treatments. In this review, we analyze the biological characteristics of RPs and emphasize their essential roles in HCC. In addition to regulating related signaling pathways such as the p53 pathway, RPs also act in proliferation and metastasis by influencing cell cycle, apoptosis, angiogenesis, and epithelial-to-mesenchymal transition in HCC. RPs are expected to unfold new possibilities for precise diagnosis and individualized treatment of HCC.

## Introduction

Ribosomal proteins (RPs) are the primary constituents of ribosomes, which are extensively distributed in various tissues. Obviously, RPs play a critical role in the process of ribosome biogenesis and protein translation [1]. Recently, increasing evidence has shown that RPs have numerous extra-ribosomal functions independent of their role in constituting ribosomes and protein biosynthesis [2]. For instance, RPs are capable of regulating p53, NF-κB, and other tumor-related signaling pathways, which are closely associated with the development of different sorts of tumors [3, 4]. Several RPs have also been identified as novel carcinogenic or tumor suppressors [5, 6].

As a primary malignant tumor, hepatocellular carcinoma (HCC) ranks the sixth most common cancer and the third leading cause of cancer-related death globally [7, 8]. The pathogenesis of HCC is complex with insidious onsets and a long incubation period. As a result, most patients with HCC are already in the advanced stage when diagnosed [9]. Currently, the clinical treatment for advanced HCC primarily relies on targeted therapy using tyrosine kinase inhibitors or systemic therapy. However, the effectiveness of these treatments is exceedingly limited [10, 11]. To date, the molecular mechanisms underlying HCC remain to be elucidated. A deeper understanding of the mechanisms will facilitate the identification of diagnostic biomarkers and effective therapeutic targets for HCC. Some studies have indicated that RPs also play an indispensable role in the progression of HCC, revealing additional potential mechanisms in HCC and guiding new possibilities for the diagnosis and treatment of HCC.

In this review, we characterize the biological characteristics of RPs and summarize recent advancements in understanding their impact on the development and drug resistance in HCC, which provides new insights for precise diagnosis and personalized treatment of HCC.

## The biological characteristics of RPs

Ribosomes are important organelles for protein synthesis in living organisms [12]. As fundamental components of ribosomes, RPs are highly conserved during evolution and extensively distributed in tissues [1]. Eukaryotic ribosome (80S) consists of the 60S large and 40S small ribosomal subunits, which comprise four ribosomal RNAs (rRNAs; 5S, 5.8S, and 28S rRNAs in the large subunit and 18S rRNA in the small subunit) and approximately 80 ribosomal proteins (RPs). The structure of the prokaryotic ribosome (70S) is similar but simpler, consisting of a 30S small subunit and a 50S large subunit. However, the categories and numbers of rRNAs and RPs are slightly distinguished: the 30S small subunit contains 16S rRNA and approximately 21 RPs; the 50S subunit consists of 5S rRNA, 23S rRNA, and roughly 31 RPs [13].

The nomenclature of ribosomal proteins is mainly in accordance with the subunits of the ribosome. In eukaryotes, the small subunit ribosomal proteins are named S1–S31, while the large subunit ribosomal proteins are designated as L1–L44 [14]. Furthermore, the large subunit has a lateral protuberance known as the ribosomal stalk, which takes part in the interaction of elongation factors with ribosome during protein synthesis [15]. The ribosomal P proteins constitute the main part of the eukaryotic ribosomal stalk, which forms a pentameric structure comprising three kinds of acidic ribosomal phosphoproteins (RPLP0, RPLP1, and RPLP2) [16].

**Fig. 1:**
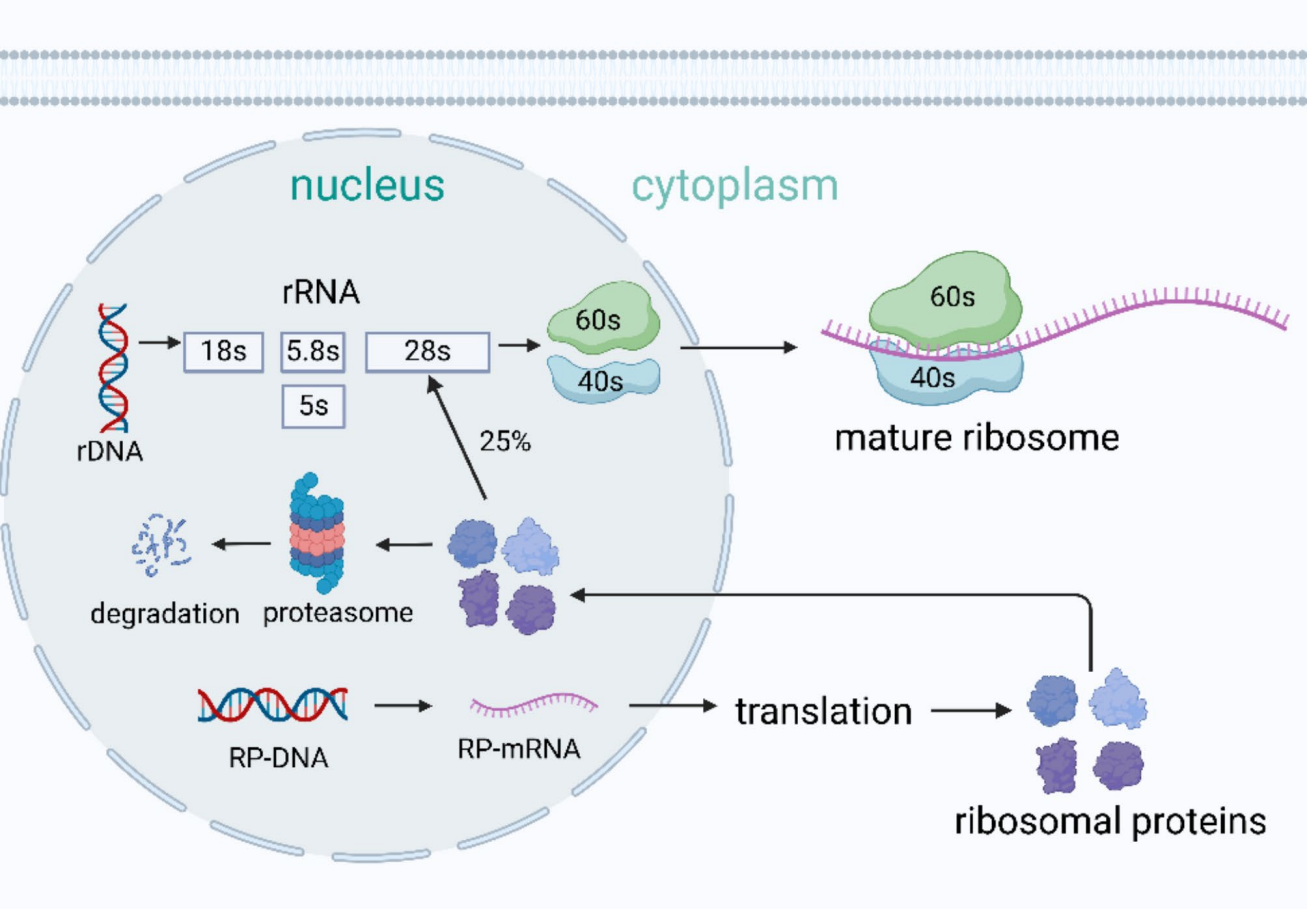
The synthesis of eukaryotic RPs. The synthesis process of eukaryotic RPs is sophisticated. The gene encoding RPs is firstly transcribed in the nucleoplasm via RNA polymerase II and then transported to the cytoplasm and translated, while the newly synthesized RPs reenter the nucleus. Merely a quarter of the RPs and rRNAs in the nucleolus assemble into ribosome subunits and transfer to the cytoplasm to form mature ribosomes, while the remaining RPs are degraded by proteasome. (Created in BioRender. Su, Q. (2024) www.BioRender.com/q85q870)

As regards the synthesis of RPs (Fig. 1), the gene encoding RPs is transcribed in the nucleoplasm via RNA polymerase II. The obtained RP mRNA is then transported to the cytoplasm for translation, and the newly synthesized RPs reenter the nucleus. Merely a quarter of the RPs and rRNAs in the nucleolus assemble into ribosome subunits and transfer to the cytoplasm to form mature ribosomes. However, the remaining RPs are degraded by proteasome [17]. This apparent energy waste of RPs overproduction and rapid degradation in the nucleolus may be attributed to the crucial role of ribosomes in various cellular processes, resulting in a certain degree of excess in synthesis [18].

Certainly, the synthesis and regulation of RPs are closely related to the growth status of the cell and the external environment, which can be regulated at multiple levels [19, 20]. Previous studies have found that most ribosomal protein genes(RPGs) in Saccharomyces cerevisiae are duplicated, whose production is primarily regulated through the splicing of introns [21]. However, research on human RPs indicated that most human RPs are encoded by single genes and widely distributed across the genome [22], which contains numerous non-functional RP pseudogenes with introns despite functional RPGs [23]. Those pseudogenes not only contribute to the study of functional RPGs but also provide evolutionary evidence as genomic landmarks [23]. Additionally, many human RPGs often share common transcription factor binding and distal regulatory regions [24, 25]. For RP mRNA, the 5’ untranslated region (UTR) of RP mRNA contains a 5’ TOP sequence, which consists of 4 to 14 pyrimidines following a cytosine [26]. This sequence, located at the 5’ end of the RP mRNA, acts as a regulatory element that can rapidly upregulate or downregulate RP levels [27]. Additionally, some RPs are involved in self-regulation. For example, ribosomal protein S13 (RPS13) could bind to the first intron of its transcript to inhibit splicing [28],while ribosomal protein S26 (RPS26) can also interact with both pre-mRNA intron I and mRNA fragments and suppress the splicing of its pre-mRNA [29]. RPs also undergo various post-translational modifications, including acetylation, methylation, phosphorylation and ubiquitination [30, 31]. As a result, the synthesis and regulation of intracellular RPs are subject to complex and dynamic regulation with both mRNA and protein levels being dynamically regulated.

In terms of functions, RPs can stabilize the structure of rRNAs and facilitate the proper folding of rRNAs to establish a functional three-dimensional structure. RPs are required to interact with rRNAs to accomplish protein synthesis. Furthermore, as scaffold proteins, RPs can not only sustain the structure of ribosomes but also regulate the spatial conformation, which plays a significant role in protein synthesis [1]. The primary site for protein synthesis is the peptidyl transferase center (PTC) within the large subunit of the ribosome [32]. Structural studies of crystals have revealed that the N-terminal tail of ribosomal protein L27 (RPL27) in bacteria stabilizes tRNA substrates in the PTC, thereby facilitating peptidyl transfer by the ribosome; while ribosomal protein L16 (RPL16) similarly enhances the binding of aminoacyl-tRNA at the A site of the ribosome [33]. Additionally, ribosomal protein L1 (RPL1), a crucial component of the ribosomal L1 stalk that interacts with the E site of the ribosome [34], binds tRNA and adjusts the conformational changes of the L1 stalk. This process aids in the release of deacylated tRNA from the ribosome, thereby completing the termination phase of translation [35, 36].

Interacting with non-ribosomal components and producing physiological effects that are not directly related to ribosome function [37], RPs can regulate gene expression, cell growth, and DNA damage repair [2]. Perturbation of RPs in mammalian cells may affect the highly ordered process of ribosomal biogenesis. For instance, the lack of RPs can lead to significant alterations in gene expression, especially at the translation level. Sometimes, it may lead to ribosomal stress, causing an imbalance of large and small subunits [38]. Downregulation of one RP may destroy a nascent subunit, leading to probable accumulation of other RPs of the subunit, occasionally with potentially profound effects such as cell death and developmental defects [39, 40]. Overexpression of RPs has been observed in certain tumors including HCC, stomach cancer, lung cancer, and breast cancer [5, 41]. Additionally, RPs participate in the regulation of p53, NF-κB, and other tumor-related pathways tied to the occurrence and development of tumors [4].

The roles of RPs in regulating p53 and its associated signaling pathways in HCC and other tumors are elaborated in the following sections of this review and will not be discussed further here. Instead, here we focus on NF-κB and additional tumor-related pathways. Firstly, it has been discovered that the polyubiquitination and degradation of RPS3 functionally suppresses the NF-κB signaling pathway, which holds promise as a therapeutic target [42]. Besides, in pancreatic cancer, collaborative down-regulation of RPL10 and NF-κB signaling pathway underlies the antiproliferative effects of dimethylaminoparthenolide (DMAPT) [43]. It is noteworthy that, apart from the later-mentioned overexpression of RPS3a which enhances HBx-induced NF-κB signaling pathway in HBV-related HCC [44], there are limited studies exploring the specific mechanisms of how other RPs influence the progression of HCC via the NF-κB signaling pathway. This may represent a direction worth exploring for future investigation. In addition to NF-κB, the proto-oncogene MYC has also been identified to be involved in ribosome biogenesis [45].In HCC, it is reported that colocalization of midline1 interacting protein 1 (MID1IP1) and c-Myc play a critical role in the progression of tumor through the regulation of RPL5 and RPL11 [46]. As discussed above, RPs are closely associated with various tumor-related pathways. This review primarily focuses on the interactions between RPs and HCC.

## Roles of RPs in the development of HCC

Recently accumulating studies have demonstrated that various RPs play their extra-ribosomal functions in HCC and contribute to the regulation of HCC development (Table 1).

Apart from being interrelated to the proverbial p53 signaling pathway, RPs participate in tremendous signaling pathways and take the shape of moldable networks to promote HCC progression by different means. For example, RPs promote the transition of cells from quiescent phase (G0 phase)/pre-DNA synthesis phase (G1 phase) and inhibit cell apoptosis, which encourages the proliferation of HCC. What’s more, RPs promote neovascularization and epithelial to mesenchymal transition (EMT), ultimately facilitating HCC metastasis.

### RPs and p53

As mentioned before, RPs have been found in numerous signaling pathways. It is worth noting that RPs exert numerous functions by directly or indirectly interacting with molecules such as p53. p53 is a tumor suppressor that has been confirmed to regulate an assortment of cellular events, including cell cycle arrest, apoptosis, and senescence [47, 48]. Generally, p53 binds to the E3 ubiquitin ligase mouse double minute 2 (MDM2, also referred to as HDM2 in human) and is then polyubiquitinated, followed by degradation in 26S proteasome [49]. On the other hand, MDM2 can precisely bind to the N-terminal domain of p53 and inhibit its transcriptional activity [50]. Conversely, MDM2 is also a target gene of p53 and is regulated by p53, thus forming a negative feedback loop between p53 and MDM2 [48].

**Fig. 2:**
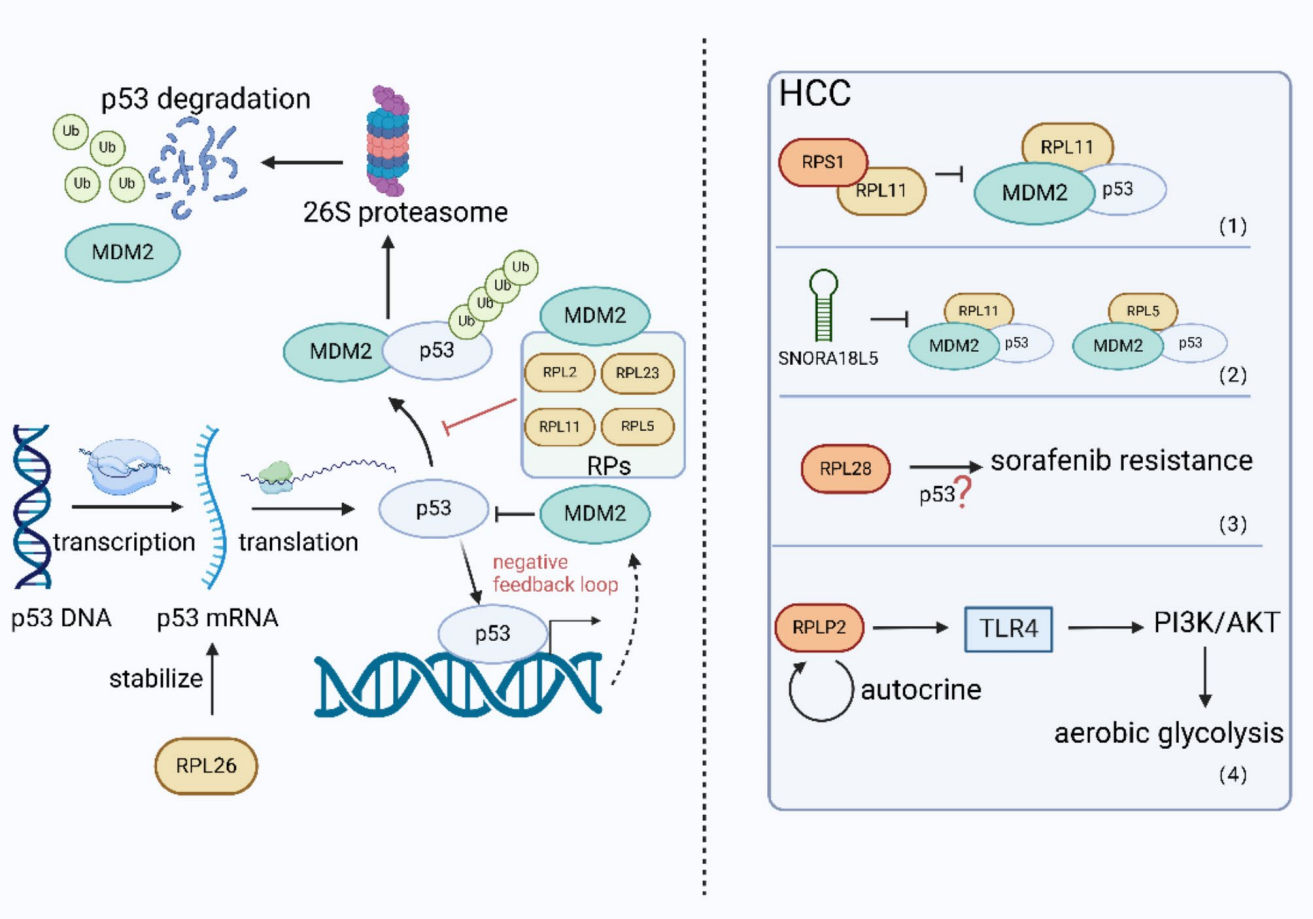
RPs interacting with p53 and MDM2. p53 is regulated by MDM2, forming a negative feedback loop. RPs can directly bind to p53 mRNA or inhibit p53 degradation by binding to MDM2. (1) The increased RPS1 binds to RPL11, thereby attenuating RPL11-MDM2-p53 signaling. (2) The expression of SNORA18L5 in liver tissues preventing RPL5 and RL11 to bind to MDM2. (3) RPL28 may inhibit programmed cell death and lead to sorafenib resistance by regulating p53. (4) RPLP2 can activate TLR4 and PI3K/AKT signaling pathways and promotes the translocation of HIF-1α into the nucleus, finally promoting aerobic glycolysis in HCC cells. (Created in BioRender. Su, Q. (2023) www.BioRender.com/z40r347)

RPs have been certified to be involved in regulating the MDM2/p53 axis with nucleolar stress, one of the key inducing events (Fig. 2) [51]. RPs can bind to the central acidic domain of MDM2 and inhibit the interaction between MDM2 and p53, thereby preventing the ubiquitination of p53 and maintaining its stability. Despite the common thread of RPs binding to MDM2, each ribosomal protein seems to have distinct binding site affinities and mechanisms [52]. The initial discovery of direct interaction with MDM2-p53 was RPL2 [53]. Subsequently, different RPs such as RPL5, RPL11, and RPL23 were recognized to block the function of E3 ubiquitin ligase in MDM2. Among them, RPL5 and RPL11 could even collaborate to inhibit the degradation of p53 [54], suggesting that RPs may shape a sophisticated network in vivo rather than functioning independently.

The RPs-MDM2-P53 axis has been identified in diversified disorders. For instance, in Diamond-Blackfan anemia, the sensitization of p53 is associated with multiple RPs (RPL5, RPL11, RPS3, RPS7, RPS27, RPS27A, and RPL23), which combine with MDM2, efficaciously rescuing p53 [55]. What’s more, the RPs-MDM2-P53 axis has also been reported in tumors without exception in HCC. One of the characteristics of HCC is the frequent alterations in the copy numbers of somatic genome [56]. Chromosome 8q gain leads to a boost in the activity of its encoded protein RPS1. The upregulated RPS1 binds to RPL11, thereby attenuating RPL11-MDM2-p53 signaling, which conversely facilitates the ubiquitination and degradation of p53 mediated by MDM2, ultimately propelling the progression of HCC [57]. The low-frequency repetition at chromosome 15q13.3 can also hyperactivate ribosomal biogenesis by increasing the expression of SNORA18L5 in liver tissues. RPL5 and RPL11 are then compelled to remain in the nucleolus, thereby preventing their binding to MDM2. This in turn increases MDM2-mediated hydrolysis of p53 and leads to cell cycle arrest, finally raising the risk for HBV-related HCC [58].

Some other RPs can even directly regulate p53 at the translation level. After DNA damage, ribosomal protein L26 (RPL26) can directly bind to the 5’ untranslated region (5’ UTR) of p53 mRNA, which enhances the binding of p53 mRNA to heavier polymers, ultimately increasing the efficiency of p53 translation and leading to an increase of p53 in protein levels [59]. It has been certified that some HCC-associated risk factors may promote DNA damage, causing genetic changes and the accumulation of genomic instability [60]. Therefore, it is worth exploring whether RPL26 plays a similar role in HCC.

Conventional local treatments for HCC such as surgical resection, radiofrequency ablation or liver transplantation commonly cease to be effective in the advanced stage [8]. Though recently targeted therapies for HCC based on tyrosine kinase inhibitors such as sorafenib, regorafenib, and other immune checkpoint modulators have presented an explosive trend [61, 62], adverse events such as diarrhea, nausea, vomiting, high blood pressure, and weight loss cannot be ignored as well [63], not to mention the teaser of drug resistance [64]. Experiments have shown that the knockout of ribosomal protein L28 (RPL28) gene significantly inhibits the proliferation of HepG2 sorafenib-resistant cells and increases cell apoptosis. Researchers then hypothesize that RPL28 may inhibit programmed cell death and lead to sorafenib resistance by regulating p53 in a similar manner, although the specific mechanism remains indistinct [65].

Previous studies have shown that p53 modulates the balance between the utilization of respiratory and glycolytic pathways through synthesis of cytochrome c oxidase 2 (SCO2) [66]. Hypoxia-inducible factor-1α (HIF-1α) ranks as the most extensively studied factors in aerobic glycolysis [67] The latest research demonstrated that acidic ribosome protein P2 (RPLP2), one of the components of the ribosomal stalk, can activate Toll-like receptor 4 (TLR4) and PI3K/AKT signaling pathways downstream through autocrine activation, which promotes the translocation of HIF-1α into the nucleus, finally promoting aerobic glycolysis in HCC cells [68]. Therefore, targeting RPLP2 may also be a potential therapeutic strategy for HCC.

### RPs and cell cycle

Cell cycle in eukaryotic cells can be normally divided into the quiescent phase (G0 phase), pre-DNA synthesis phase (G1 phase), DNA synthesis phase (S phase), late DNA synthesis phase (G2 phase), and cell division phase (M phase) [69]. These processes are coordinated by a complicated network of interactions among proteins, enzymes, cytokines, and signaling pathways. Cell cycle is essential for cell proliferation, growth, and repair, thus it is conspicuous to consider the correlation between the development and metastasis of tumors and cell cycle [70, 71].Therefore, cell cycle arrest is condignly one of the significant targets of antitumor agents [72].

**Fig. 3:**
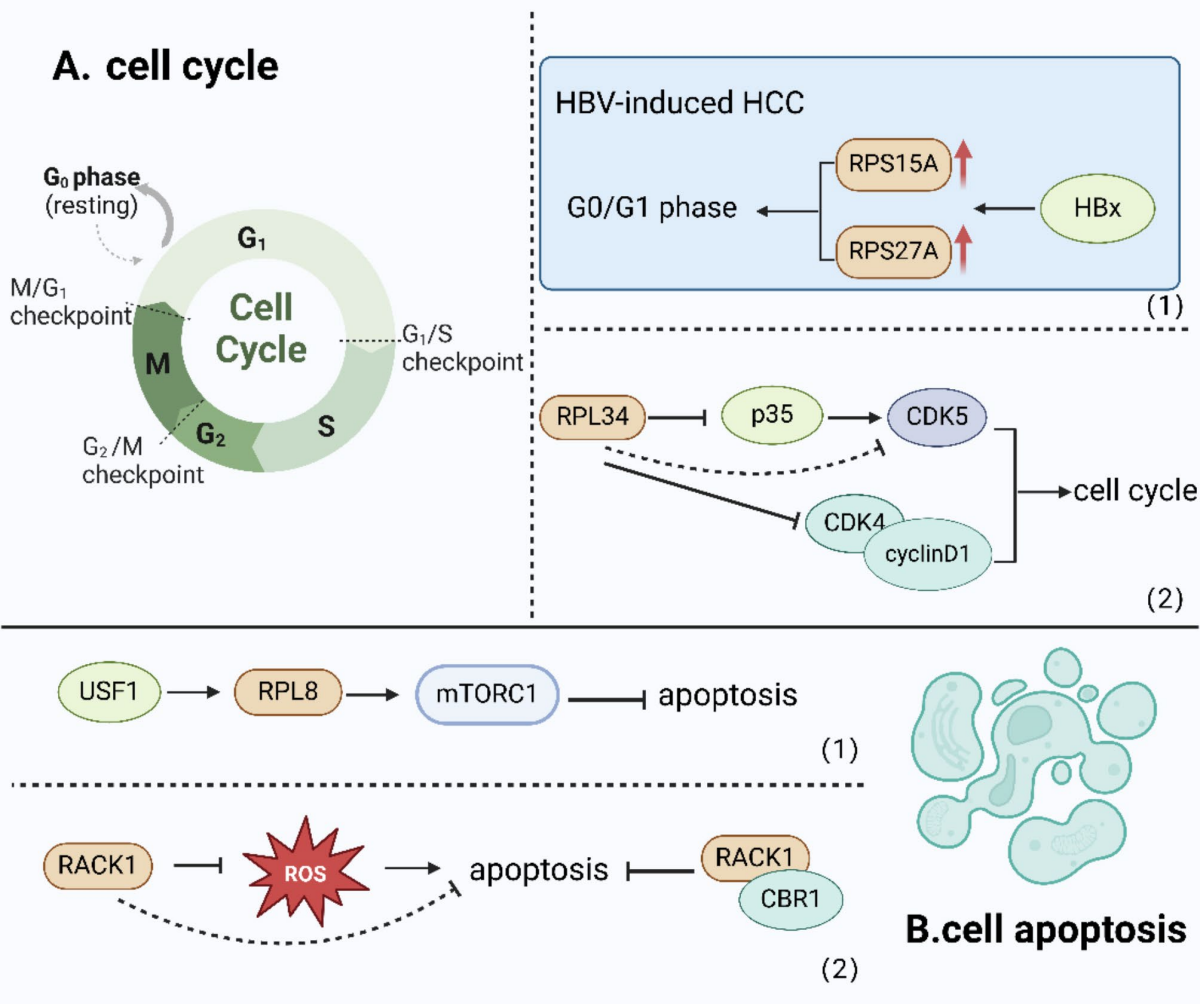
RPs and proliferation of HCC. A. RPs influence cell cycle. (1) In HBV-induced HCC, RPS27A and RPS15A are upregulated by HBx and may influence the progression of HCC at G0/G1 phase. (2) RPL34 directly interacts with CDK4 while inhibiting CDK4/cyclin D1 activity and indirectly inhibits CDK5 by effectively inhibiting the activity of p35 which activates CDK5. B. RPs influence apoptosis. (1) RPL8 is regulated by USF1 and activate the mTORC1 signaling pathway. (2) RACK1 inhibits the production of ROS and protects HCC cells from TNF-α-induced cell death through its interaction and regulation with CBR1. (Created in BioRender. Su, Q. (2022) www.BioRender.com/p62r405)

As early as 1999, ribosomal protein L34 (RPL34) was identified as an interacting protein of cyclin-dependent kinases 4 and 5 (CDK4 and CDK5). RPL34 interacts with CDK4 and inhibits CDK4/cyclin D1 activity. Nevertheless, RPL34 does not directly interact with CDK5, which effectively inhibits the activity of p35, the protein that activates CDK5, and thus indirectly inhibits CDK5 [73]. Several studies have also reported that silencing certain RPs in HCC can forcibly arrest the cell cycle of HCC cells and inhibit cell proliferation. Hepatitis B virus (HBV) infection ranks as one of the prominent etiologies of HCC [74]. Simultaneously, the hepatitis B virus X protein (HBx) contributes to the development of HCC [75]. As a pleiotropic transactivator, HBx stimulates a wide range of viral and cellular promoters [76]. Ribosomal protein s27a (PRS27A) and ribosomal protein s15a (PRS15A) are both upregulated in HBV-induced HCC patients. Silencing both RPs has been verified to vigorously inhibit the cell cycle of HBX-transfected HCC cell lines at the G0/G1 phase, thereby inhibiting the proliferation of HCC. The presence of HBx can trigger the promoter of RPS27A and sensibly foster the expression of endogenous RPS27A, which contributes to maintaining the cell size of liver cancer cells as they propagate [77]. RPS15A is also regulated by the HBx likewise, which peculiarly targets the RPS15A gene by intensifying the TGF-β signaling pathway [78] (Fig. 3).

### RPs and apoptosis

Apoptosis refers to a self-regulated process of programmed cell death that is administered by genes [79]. Under the regulation of apoptins and anti-apoptins, the body can systematically eliminate damaged cells and maintain homeostasis [80], hence one of the most crucial indicators of tumors is the dysregulation of apoptotic cell death mechanisms [81]. Apoptosis disorders not only interrelate with the occurrence and development of tumors, but also induce resistance to treatment [82]. That’s why investigating mechanisms and molecules associated with apoptosis can help tackle drug resistance.

The expression of ribosomal protein L8 (RPL8) is elevated in HCC and is regulated by upstream transcription factor 1 (USF1), which can activate the mTORC1 signaling pathway. The reverse experimental results also support this conclusion, as evidenced by the significant decrease in the levels of p-mTOR/mTOR and p-RPS6KB1/RPS6KB1 upon silencing of RPL8, which hastens apoptosis and diminishes the metastasis and invasion of HCC. Consequently, RPL8 may affect HCC progression by regulating the mTORC1 signaling pathway [83]. Another RP called ribosomal protein receptor for activated C kinase 1 (RACK1) was originally recognized as binding and activating protein kinase C [84, 85]. However, further research has revealed that RACK1, as a component of the 40 S subunit of the ribosome, exhibits a diverse range of functions, serving as a scaffold protein for multitudinous kinases and receptors [86]. It has been confirmed that the expression of RACK1 is upregulated in HCC. RACK1 inhibits the production of reactive oxygen species (ROS) and protects HCC cells from TNF-α-induced cell death through its interaction and regulation with carbonyl reductase 1 (CBR1) [87]. Simultaneously, the high level of O-linked β-N-acetylglucosamine (O-GlcNAc) at Ser122 in RACK1 can enhance its stability, which is also a key mediator in connecting O-GlcNAc metabolism with cap-dependent translation during HCC tumorigenesis [88]. Furthermore, RACK1 is also involved in the phosphorylation of eukaryotic initiation factors (eIFs) via PKCβII, the activation of which is relevant to the progression of diverse tumors. Nevertheless, further research is indispensable to ascertain whether RACK1 promotes the development of HCC through this pathway as well [89] (Fig. 3).

### RPs and angiogenesis

Angiogenesis is an elaborate biological process that involves the formation of new blood vessels in response to physiological and pathological conditions, which promotes the migration and invasion of cancer cells [90]. Studies have indicated that abnormal angiogenesis in HCC promotes hepatocyte development, migration, and invasion [91, 92].

**Fig. 4:**
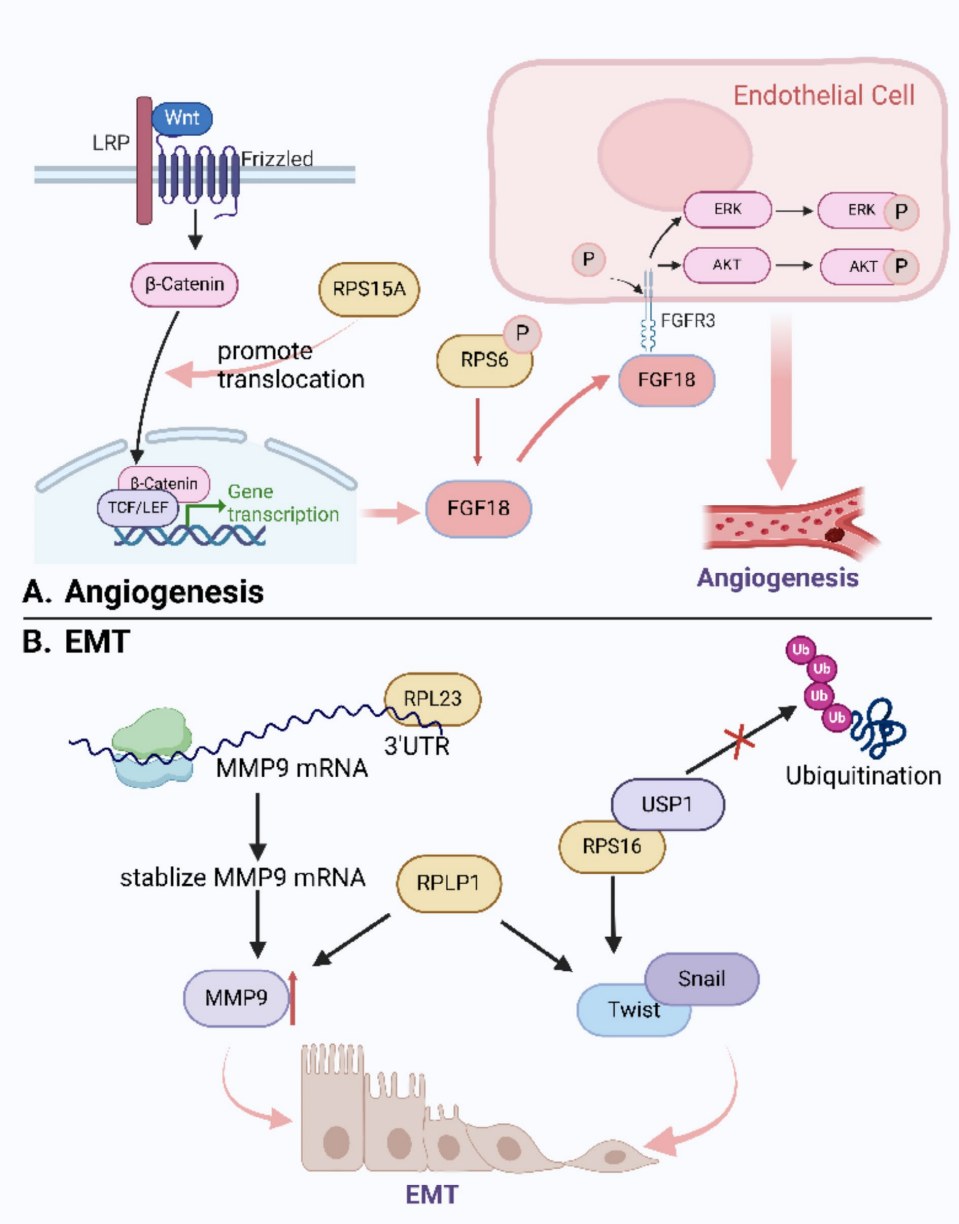
RPs and metastasis of HCC. **A.** RPs influence angiogenesis. RPS15A promotes β-catenin translocation into the nucleus and upregulate the expression of fibroblast growth factor 18 (FGF18). Phosphorylation of RPS6 also participates in the activation of FGF18. FGF18 then interacts with FGFR3, activates the AKT and ERK signaling pathways and enhances the potential of angiogenic in endothelial cells in HCC. **B.** RPs influence EMT. RPL23 binds to the 3’UTR of MMP9 to enhance its mRNA stability and increase the expression of MMP9, promoting metastasis of HCC by EMT effectively. RPLP1 may equally contribute to the induction of EMT. USP1 deubiquitinates and stabilizes RPS16 through its DUB activity, which promotes the expression of transcription factors such as Twist and Snail. (Created in BioRender. Su, Q. (2023) www.BioRender.com/j04s369)

Several RPs are traced to mediate angiogenesis in HCC (Fig. 4). RPS15A, apart from the mentioned ability to strike the cell cycle as mentioned above, is firmly associated with the microvascular density of HCC [78, 93]. In HCC, RPS15A enhances the activation of the Wnt signaling pathway and consequently promotes β-catenin translocation into the nucleus. The expression of fibroblast growth factor 18 (FGF18) within the tumor microenvironment then ramps up after the activity of the β-catenin and T cell factor/lymphoid enhancer-binding factor (Tcf/Lef) up-regulated. Additionally, the phosphorylation of RPS6 also participates in the activation of FGF18 [94]. FGF18 interacts with its receptor, FGFR3, located on endothelial cells, thereby cascade activating the AKT and ERK signaling pathways and enhancing the potential of angiogenic in endothelial cells in HCC [93]. Furthermore, RPS6 facilitates fat synthesis through the AKT-mTORC1-RPS6 pathway at both transcriptional and post-transcriptional levels, including inhibition of fatty acid synthase (FASN) ubiquitination by the USP2a de-ubiquitinase and disruption of the sterol-regulatory element binding proteins (SREBP) 1 and SREBP2 degradation complexes. Abnormal fat synthesis equally accelerates the development of HCC [95] (Fig. 4).

### RPs and epithelial to mesenchymal transition

The epithelial-to-mesenchymal transition (EMT) is a complex process in which specific conditions can disrupt the tight intercellular junctions between epithelial cells, leading to the loss of their inherent polarity, organization, and consistency [96]. EMT involves diverse genes and signaling pathways that result in decreased cell adhesion and increased migration and invasion [97]. EMT generally occurs during embryonic development and has recently been found to be necessary for both local and distant transformation progression of malignancies, including HCC [98]. This process entails the involvement of multiple RPs (Fig. 4).

Ribosomal protein L23 (RPL23) exhibits a dominant upregulation in metastatic HCC tissues and is positively correlated with decreased survival rates among HCC patients. Existing studies indicated that RPL23 binds to the 3’UTR of matrix metalloproteinase 9 (MMP9) to enhance its mRNA stability and increase the expression of MMP9 at the post-transcriptional level [99]. MMP9 belongs to the matrix metalloproteinases family mediating extracellular matrix degradation and is tightly associated with HCC metastasis [98]. Thus, the behavior of RPL23 in increasing MMP9 expression by stabilizing mRNA effectively promotes metastasis of HCC through EMT. Another study suggested that the overexpression of RPL23 can induce cisplatin resistance in epithelial ovarian cancer (EOC) cell lines A2780 and SKOV3 by inducing EMT [100]. Platinum-based antitumor drugs have broad-spectrum antitumor activity and are popularly applied in the treatment of over 80% of tumors [101]. In HCC, cisplatin is often administered intravenously as a chemotherapy treatment while cisplatin resistance often occurs [102]. Further research is needed to determine whether RPL23 also plays a role through the same mechanism as in EOC. If so, RPL23 may be a potential target for addressing cisplatin resistance in HCC.

Another ribosomal protein LP1 (RPLP1) is up-regulated in HCC as well and is significantly relevant to poorer prognosis in patients with HCC. Silencing RPLP1 decreases the levels of vimentin, Snail, Slug, N‑cadherin, MMP‑2, and MMP‑9, while increasing the levels of β‑catenin, E‑cadherin, claudin‑1, and tissue inhibitors of metalloproteinase-1(TIMP‑1), which are all essential components linked to EMT, eventually inhibit proliferation of HCC [103]. These findings suggest that RPLP1 may equally contribute to the induction of EMT. A previous study on colorectal cancer found that RPLP1 deficiency resulted in the accumulation of reactive oxygen species (ROS) and the activation of the MAPK1/ERK2 signaling pathway in colon cancer cells. However, the detailed mechanism remains unclear [104]. Future studies need to be performed to investigate the role of RPLP1 in HCC progression.

As a substrate of ubiquitin-specific peptidase 1 (USP1), ribosomal protein S16 (RPS16) can be rigorously regulated by ubiquitin-proteasome pathways. USP1 recognizes and binds to RPS16 through its C-terminal (401-785aa). Then USP1 deubiquitinates and stabilizes RPS16 through its deubiquitinating enzyme (DUB) activity, which promotes the expression of transcription factors such as Twist and Snail [105]. Twist and Snail can regulate downstream genes through distinct mechanisms, thereby leading to EMT and contributing to HCC [106, 107].

## Roles of RPs in diagnosis and prognosis of HCC

HCC is recognized as one of the most common malignant tumors in the world, characterized by its insidious onset and long incubation period. It is not a surprise that the majority of patients are typically diagnosed at an advanced stage [9]. Therefore, early diagnosis of HCC is of utmost importance to facilitate effective treatment and life extension [108]. But it is a huge challenge. The early diagnosis previously depended primarily on ultrasound monitoring (US) and alpha-fetoprotein (AFP); however, the sensitivity and specificity of these methods were deemed unsatisfactory [109]. Researchers have discovered novel imaging techniques and non-invasive biomarkers with good specificity and sensitivity which have the potential to diagnose late-stage HCC [110]. Based on studies on the relationships between RPs and HCC, RPs are expected to become novel biomarkers for HCC diagnosis and contribute to predicting prognosis of HCC (Table 1). However, it is worth noting that the mRNA and protein expression levels of the RPs and MRPs listed in the table were derived from analyses of HCC cells in a specific state. As mentioned earlier, the synthesis and regulation of RPs are highly complex, and the mRNA and protein levels of RPs within cells are expected to fluctuate dynamically. Therefore, data taken from databases should be considered with caution regarding their reliability.

As early as 2011, researchers applied immunohistochemistry to analyze the expression of ribosomal protein L36 (RPL36) in 60 specimens from HCC patients and assessed the prognostic value of RPL36 through univariate and multivariate analysis of patient survival. The data indicate that RPL36 is a promising biomarker for predicting the prognosis of HCC despite limited cases [111]. Subsequently, with the rapid advancement of bioinformatics and high-throughput research methods, several research teams have identified RPs with diagnostic and prognostic potential through weighted gene co-expression network analysis using datasets. For example, ribosomal protein S8 (RPS8) can serve as a novel biomarker for alcohol-related hepatocellular carcinoma [112], while ribosomal proteins such as RPL19, RPS7, RPS14, RPS24, RPS3A, RPS27, RPS36, RPL32, and RPL11 have been identified as early diagnostic and prognostic markers for common hepatocellular carcinoma [112–116].

Recently, there exist some emerging theories suggesting that some RPs may be associated with immune escape in HCC which deserves attention. Firstly, ribosomal protein S24 (RPS24) is proven to promote cell proliferation and the formation of an immunosuppressive microenvironment in HCC [117]. Single sample gene set enrichment analysis (ssGSEA) and immunohistochemistry also exhibited a strong negative correlation between ribosomal protein S3a (RPS3A) expression and the infiltration of tumor immune cell [114]. In addition, RPS3A also interacts with HBx protein through N-terminal domain to enhance the expression of intracellular soluble HBx protein, which then activates HBX-induced NF-κB signaling pathway and enhances the possibility of HBV-induced tumor development [44].

Nevertheless, all the above are mainly based on bioinformatics analysis for speculation, and only a small number of in vivo experiments have been conducted to assist in proving which is not sufficient to prove the decisive role of RPs in the diagnosis and prognosis of HCC, thus further research is still needed to confirm.

## Mitochondrial ribosomal proteins in HCC

There exists a characteristic organelle in eukaryotes named mitochondria, which is hailed as “power factory” and is evolutionarily conserved that mammals acquired from alphaproteobacteria through the process of endosymbiosis [118]. Mitochondria have their own ribosomes that can synthesize a handful of proteins [119]. What we mentioned earlier in this review, more accurately, should be referred to as cytoplasmic ribosomal proteins (CRPs). This part we will focus on the role of mitochondrial ribosomal proteins (MRPs) in HCC (Table 1).

Mitochondria reserves the coding sequences of 37 genes during evolution, which encode 13 proteins involved in cellular energy metabolism [120]. The mitochondrial translation machine consists of tRNAs and 55S mitochondrial complexes, which comprise of a large 39S subunit involved in catalyzing the peptidyl-transferase reaction, and a small 28S subunit providing the platform for mRNA binding and decoding [121]. The 39S subunit is composed of 16S mitochondrial rRNA (mt-rRNA) and 50 MRPs, whereas the 28S subunit consists of 12S mt-rRNA and 29 MRPs [122]. The MRPs are encoded by the nuclear genome. Once transcribed, the corresponding mRNAs are transported on the cytoskeleton to localize on ribosomes in the proximity of the mitochondria and then, the nascent MRPs are imported into the organelle through the outer membrane transposase (TOM) and inner membrane transposase (TIM) [123]. The unassembled copies of MRPs that are not involved in mitochondrial assembly are degraded to avoid excessive accumulation in organelles [119].

An increasing number of studies indicate that MPRs are not only involved in mitochondrial oxidative phosphorylation but also closely related to various diseases. In a bioinformatics analysis, researchers found 14 MRP genes, including MRPS21, MRPS23, MRPL9, DAP3, MRPL13, MRPL17, MRPL24, MRPL55, MRPL16, MRPL14, MRPS17, MRPL47, MRPL21, and MRPL15 were significantly upregulated differentially expressed genes (DEGs) in HCC tumor samples in comparison to normal samples. Receiver-operating characteristic curve analysis also indicated that all 14 DEGs show good diagnostic performance [124].

**Fig. 5:**
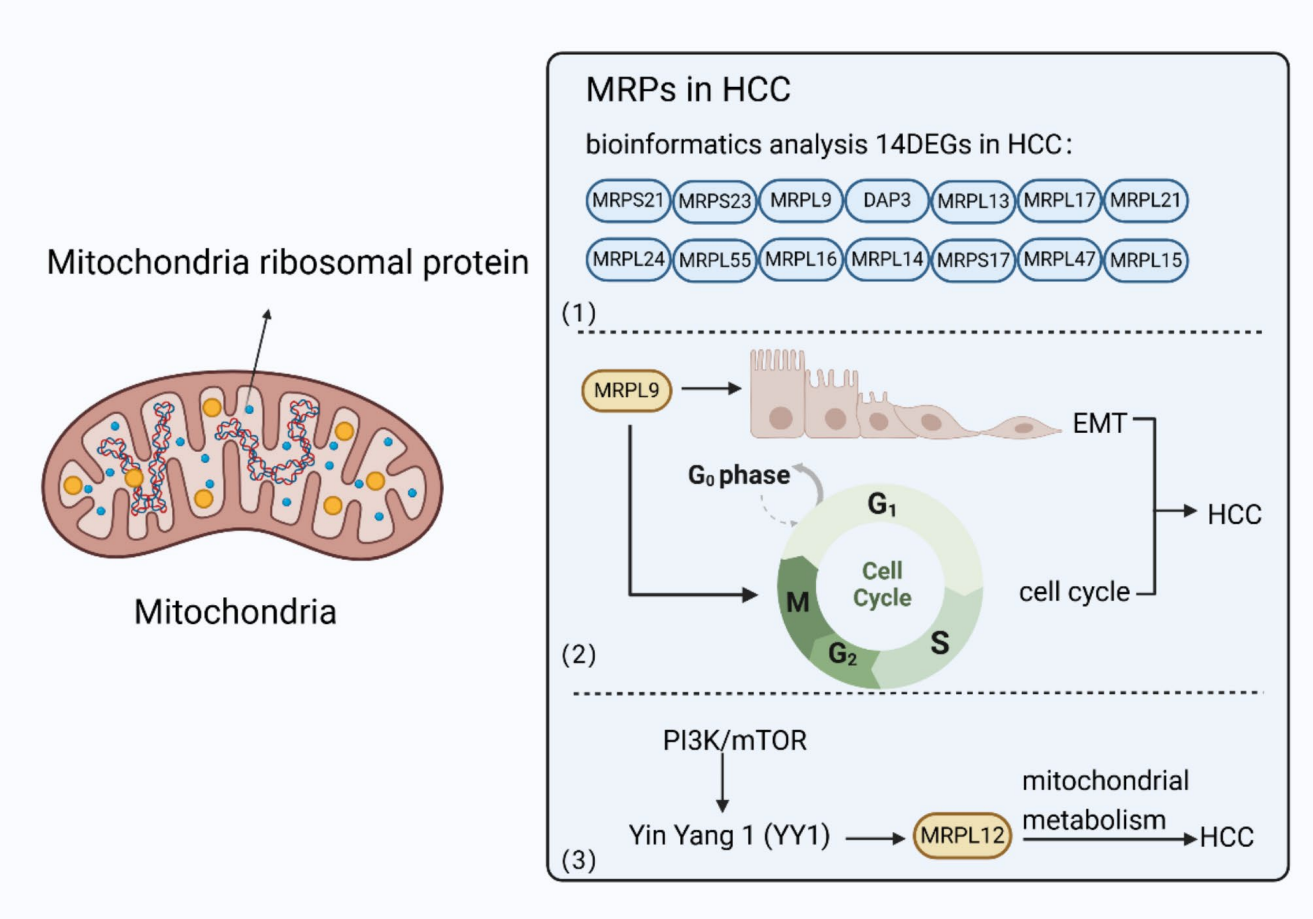
MRPs in HCC. Mitochondria have their own MRPs. (1) In a bioinformatics analysis, researchers found 14 MRP genes including MRPS21, MRPS23, MRPL9, DAP3, MRPL13, MRPL17, MRPL24, MRPL55, MRPL16, MRPL14, MRPS17, MRPL47, MRPL21, and MRPL15 were significantly upregulated DEGs in HCC tumor samples in comparison to normal samples. (2) MRPL9 can accelerate the progression of EMT and advance the transition of G1/S phase in HCC. (3) MRPL12 participates in the progression of HCC by regulating mitochondrial metabolism. MRPL12 knockdown could attenuate the YY1 overexpression or PI3K/mTOR activation-induced malignant phenotype in HCC cells. (Created in BioRender. Su, Q. (2023) www.BioRender.com/h84m059)

Studies focusing on concrete MRPs have also revealed the roles of certain MRPs in HCC. In vitro experiments have found that upregulated MRPL9 can significantly promote tumor proliferation, metastasis, and interfere with the cell cycle by advancing the transition of G1/S phase. MRPL9 can also accelerate the progression of EMT, which is crucial in the early stage of HCC metastasis [125]. Mitochondrial dysfunction and metabolic reprogramming are the main characteristics of HCC [126, 127]. Inhibiting oxidative phosphorylation (OXPHOS) effectively alleviated the tumor-promoting effect caused by overexpression of MRPL12, indicating that MRPL12 participates in the progression of HCC by regulating mitochondrial metabolism. Yin Yang 1 (YY1) has been identified as a transcription factor responsible for regulating MRPL12, while the PI3K/mTOR pathway was found to act as an upstream regulator of YY1. MRPL12 knockdown could attenuate the YY1 overexpression or PI3K/mTOR activation-induced malignant phenotype in HCC cells [128]. These results all highlight the possibility of targeting MRPs as promising therapeutic strategies for the treatment of HCC (Fig. 5).

## RPs and MRPs in the treatment of HCC

Currently potentially curative treatment modalities for early and intermediate HCC comprise liver resection, liver transplantation and local destruction methods such as radiofrequency ablation. While sorafenib, a multikinase inhibitor, has been established as the standard systemic therapy for patients who are in the advanced stage [129]. However, HBV-HCC may be more applicable for employing immune checkpoint inhibitor therapy or a combination of immune checkpoint inhibitors and targeted drugs [130]. Among them, immunosuppressants targeting programmed cell death 1 (PD-1) and its ligands have always been a hotspot. Surprisingly, HCC-PD-1 can physically bind with RPS6 and promote its phosphorylation, which promotes the progression of HCC [131]. RPs closely related to tumor immunity suggest a new possibility of immunotherapy. Research has identified that RPL15 is a novel target protein of Topotecan ((S)-9-dimethylaminomethyl-10-hydroxycamtothecin hydrochloride, TPT) using a mouse melanoma tumor model. TPT, a semi synthetic analogue of camptothecin, is an early topoisomerase I inhibitor. When combined with RPL15, TPT not only inhibits the interaction between RPL15 and RPL4 but also reduces the stability of RPL4, eventually promoting the secretion of damage-associated molecular patterns (DAMPs) and contributing to antitumor immune activation [132]. RPL15 also acts in the progression of HCC through the p53-MDM2 signaling pathway and EMT [133]. However, the combination of TPT and cisplatin seems to be ineffective for patients with advanced HCC in a phase II study [134]. It is expected to develop analogues of camptothecin with lower toxicity and target RPL15 for the treatment of HCC.

Except for RPL15, targeting each upregulated RPs or MRPs in HCC cells may become a therapeutic direction for HCC. Nevertheless, currently there exist some confusing issues: firstly, searching the DrugBank database reveals that the known potential drugs targeting RPs or MRPs mentioned in this review are not yet sufficiently understood (Table 2). Among those, (S)-3-phenyllactic acid, Anisomycin and Puromycin are still under the experiment. Though Copper and Artenimol also target some RPs, current researches for both drugs mainly focus on their effects on other targets: Novel copper complexes is expected to demonstrate their skills in tumor treatment [135]. But Artenimol tend to be more effectively applied in the field of malaria as an artemisinin derivative according to the description of Drugbank.

Secondly, it is worth considering that if directly targeting one or more RPs systematically, the ribosome function of normal cells may also be inhibited, which may affect the ordinary protein synthesis. As eukaryotic cells adjust the number of ribosomes per cell based on growth rate, in rapidly growing non-tumor cells, most RPs are upregulated to meet the increased demand for protein synthesis as a result of heightened metabolic activity [136]. Notably, in these rapidly growing non-tumor cells, RPs also exert their ubiquitous extra-ribosomal functions and participate in the regulation of multiple signaling pathways [137]. Therefore, if RPs are not differentially expressed between those rapidly growing non-tumor cells and HCC cells, they are unlikely to serve as targets for chemotherapeutic intervention. Thus, it is necessary to identify characteristic HCC cells and target those elevated RPs in tumor cells. But how to deliver drugs specifically to HCC cells remains a challenge.

Perhaps emerging nanoparticle technologies have the power to solve the puzzle. Among them, exosomes, as endogenous extracellular vesicles with lipid bilayer membranes, are considered as a new generation of natural nanoscale delivery systems [138]. In vivo and in vitro experiments have evidenced that exosomes from adipose tissue-derived mesenchymal stem cells (AMSCs) effectively convey MiR-199a-3p (miR-199a) to HCC cells and elevate their chemotherapy sensitivity [139]. In order to better target cells and improve their constancy in vivo, exosomes can also be modified appropriately [140]. For example, efficient surface labeling techniques can be used to manufacture monoclonal antibody exosomes: the SSTR2 mAb-exosomes can deliver romidepsin to neuroendocrine cancer cells [141, 142]. Some researchers hypothesized that modifying the surface of HCC exosomes expressing chemokine receptors and loading with anti-tumor drugs may also enhance the chemotaxis of exosomes towards HCC and achieve the goal of targeted therapy [143].

Therefore, modified exosomes have extraordinary potential in the treatment of HCC. There is hope to load drugs that may target RPs into modified exosomes and selectively deliver them to HCC cells, thereby inhibiting the occurrence and development of HCC. This may also be a promising treatment direction for HCC.

Furthermore, RPs may participate in treatment decision and resistance in relation to tumor stage or grade. For instance, as previously mentioned, upregulated MRPL12 has been correlated with advanced tumor stage, higher tumor grade, and poor prognosis in HCC [128]. Looking ahead, MRPL12 could potentially serve as a complementary factor in guiding treatment decisions for HCC in conjunction with tumor stage or grade. In terms of drug resistance, RPs have been implicated in resistance to a range of antibiotics and influence the synthesis and overexpression of multidrug resistance genes [2, 144, 145]. Real-time quantitative reverse transcription PCR (RT-qPCR) was used to measure the total RNA in amycin-resistant and susceptible HepG2 cells. The results indicated that the transcription level of RPL24 was 7.7-fold higher in amycin-resistant HepG2 cells compared to susceptible cells. Additionally, the expression of RPL24 contributed to increased drug resistance in susceptible cells [146]. RPL4 and RPL5 have also been found to be overexpressed in doxorubicin resistant human CRC cell line LoVoDxR [147]. Characterizing more RPs associated with drug resistance in HCC could aid in overcoming resistance to a broader range of antitumor agents.

## Summary and outlook

In summary, as the main components of ribosomes, RPs are not only involved in protein synthesis but also participate in the occurrence and development of various cancers, such as HCC. RPs up-regulated in HCC contribute to the proliferation and invasion of HCC through intricate mechanisms. It is expected for RPs to become novel potential biomarkers for the early diagnosis of hepatocellular carcinoma. RPs also have the potential to solve the problems of drug resistance and become new targets for future therapy in HCC according to the listed evidence.

However, there are still some challenges that need to be addressed. Firstly, it is worth noting that there are numerous types of RPs, the mechanisms of the vast majority of which in HCC are still not fully understood and require further research. The specific mechanisms of limited RPs associated with HCC also remain a mystery worth exploring. Although bioinformatics is highly developed nowadays, with methods such as weighted gene co-expression network analysis, researchers have found that certain RPs are promising emerging biomarkers for the diagnosis and prognosis of HCC, the timeliness, specificity, and sensitivity of RPs for HCC still need to be repeatedly proved by specific clinical application. Another question worth raising is whether inhibiting one or more RPs would affect the protein translation and physiological function of normal cells if targeting RPs is truly applied in the future. Perhaps with the advancement of technology, using exosomes to specifically deliver drugs targeting RPs and MRPs may one day become a reality. These are all issues that warrant consideration and solution. Similarly, we barely have a smattering of knowledge of the role of MRPs in HCC. Future research on the relationship between mitochondria and metabolism in HCC may uncover more mysterious aspects of MRPs located in mitochondria. There are so many mysteries surrounding RPs and MRPs in HCC and other diseases, awaiting our discovery and exploration.

In conclusion, although research on RPs and MRPs in HCC still stands in an initial stage, it is foreseeable that targeting these proteins will play a unique role in the precision diagnosis and personalized therapy of HCC. That is to say, the prospect of application for them in HCC is extensive and worth pursuing.

**Table 1:** Some RPs and MRPs in HCC

RPs/MRPs	mRNA expression in HCC	Protein expression in HCC	HR (OS)	Possible mechanisms and roles in HCC	Refs
RPL11	↓ (0.84)	↑ (0.041)	1.06	Inhibit the degradation of p53; An early diagnostic and prognostic marker	[54, 58]
RPL5	↑ (1.18)	↑↑ (0.534)	1.36	Inhibit the degradation of p53	[58]
RPL28	↑ (1.45)	↓ (-0.233)	0.99	Regulate p53	[65]
RPLP2	↑↑ (1.38)	↓↓ (-0.517)	1.06	Promote aerobic glycolysis	[68]
RPL34	↓ (0.90)	↑↑ (0.637)	0.79	Inhibit CDK4/cyclin D1 and CDK5	[73]
RPL8	↑↑↑ (2.06)	↑ (0.222)	1.29	Regulate the mTORC1 signaling pathway	[83]
RACK1	↑ (1.23)	↑↑↑ (1.487)	1.58	Inhibit the production of ROS; Connect with cap-dependent translation	[87, 88]
RPS6	↑ (1.09)	↑↑ (0.768)	1.28	Participate in the activation of FGF18; Facilitate fat synthesis through the AKT-mTORC1-RPS6 pathway	[94, 95]
RPL23	↑ (1.31)	↑↑ (0.546)	0.83	Increase MMP9 expression	[99]
RPLP1	↑ (1.46)	↑ (0.175)	1.16	Contribute to the induction of EMT	[103]
RPS16	↑ (1.25)	↓ (-0.279)	1.15	Regulated by USP1; Promote the expression of Twist and Snail	[105]
RPL36	↑ (1.22)	↓ (-0.447)	1.12	A promising biomarker	[111]
RPS8	↑ (1.29)	↓ (-0.012)	1.37	A novel biomarker for alcohol-related HCC	[112]
RPL19	↑ (1.19)	↓↓ (-0.682)	1.05	An early diagnostic and prognostic marker	[113]
RPS7	↑ (1.31)	↑ (0.147)	1.16	An early diagnostic and prognostic marker	[112]
RPS14	↑ (1.33)	↑ (0.166)	1.12	An early diagnostic and prognostic marker	[112]
RPS24	↑ (1.21)	↓ (-0.372)	1.08	Promote cell proliferation and the formation of an immunosuppressive microenvironment; An early diagnostic and prognostic marker	[40, 117]
RPS3A	↑ (1.15)	↓ (-0.258)	1.12	Negatively correlated with infiltration of tumor immune cell; Interacts with HBx protein; An early diagnostic and prognostic marker	[44, 114]
RPS27	↑ (1.40)	↑ (0.356)	0.66	An early diagnostic and prognostic marker	[77, 112]
RPL32	↑ (1.12)	↑↑ (0.551)	1.12	An early diagnostic and prognostic marker	[115]
MRPS21	↑↑↑ (2.09)	↑↑↑ (1.054)	1.40	DEGs in HCC	[124]
MRPS23	↑↑ (1.73)	↑↑↑ (1.222)	1.91	DEGs in HCC	[124]
MRPL9	↑↑ (1.66)	↑↑↑ (1.239)	1.51	DEGs in HCC; Accelerate the progression of EMT	[124, 125]
DAP3	↑↑ (1.78)	↑↑↑ (1.307)	1.72	DEGs in HCC	[124]
MRPL13	↑↑ (1.68)	↑↑↑ (1.333)	1.13	DEGs in HCC	[124]
MRPL17	↑ (1.25)	↑↑↑ (1.466)	1.22	DEGs in HCC	[124]
MRPL24	↑↑ (1.96)	↑↑↑ (1.758)	1.05	DEGs in HCC	[124]
MRPL55	↑↑ (1.62)	↑↑ (0.915)	0.84	DEGs in HCC	[124]
MRPL16	↓ (0.87)	↑↑↑ (1.729)	0.81	DEGs in HCC	[124]
MRPL14	↑ (1.19)	↑↑↑ (1.288)	0.94	DEGs in HCC	[124]
MRPS17	↑↑ (1.65)	↑↑ (0.887)	1.22	DEGs in HCC	[124]
MRPL47	↑ (1.23)	↑↑↑ (1.739)	1.07	DEGs in HCC	[124]
MRPL21	↑ (1.22)	↑↑↑ (1.525)	0.90	DEGs in HCC	[124]
MRPL15	↑ (1.34)	↑↑↑ (1.445)	1.12	DEGs in HCC	[124]
MRPL12	↑ (1.24)	↑↑↑ (1.869)	1.04	Regulate mitochondrial metabolism	[148]

Table 1 lists some RPs and MRPs in HCC mentioned in this review. The Gene Set Cancer Analysis (GSCA) database (https://guolab.wchscu.cn/GSCA/#/; accessed on January 15th, 2024) was used to identify differential expression and survival analysis of various RPs and MRPs in HCC [149, 150]. Protein level data available from the National Cancer Institute’s Clinical Proteomic Tumor Analysis Consortium (CPTAC) were analyzed using the UALCAN tool (https://ualcan.path.uab.edu; accessed on January 15th, 2024) [151–154] to measure their expression of protein in HCC

For the column of mRNA expression in HCC and protein expression in HCC, ↑, ↑↑, ↑↑↑, ↓, ↓↓, ↓↓↓ indicate different levels of change. For mRNA expression in HCC, ↑ represent fold change (FC) is between 1 and 1.5; ↑↑ represent FC is between 1.5 and 2; ↑↑↑ represent FC is greater than 2; ↓ represent FC is less than 1. FC greater than 1 indicates an increase in mRNA expression in HCC, while conversely, the expression decreases. For protein expression in HCC, the values in parentheses represent the absolute difference between the median of z-values of protein expression levels: ↑: 0-0.5; ↑↑: 0.5-1; ↑↑↑: >1; ↓: -0.5-0; ↓↓: -1–0.5. The column of HR (OS) lists concrete Hazard ratio (HR) in overall survival (OS)

Notably, the synthesis and regulation of RPs is complicated and dynamic. Therefore, these data must be considered with care

**Table 2:** Some drugs targeting RPs

Drugbank ID	Name of drug	Drug Groups	Drug type	Targets
DB02494	(S)-3-phenyllactic acid	Experimental	Small Molecule	RPL11, RPL8, RPL23, RPL19
DB07374	Anisomycin			RPL11, RPL8, RPL23, RPL19
DB08437	Puromycin			RPL11, RPL8, RPL23, RPL19
DB09130	Copper	Approved, Investigational		RACK1
DB11638	Artenimol	Approved, Experimental, Investigational		RPS6, RPS8

Table 2 lists some drugs targeting RPs. The DrugBank database (https://go.drugbank.com/; accessed on February 5th, 2024) was used. Other RPs and MRPs that appeared in the main text of this review but are not listed here were not retrieved from Drugbank for corresponding drugs

## Data Availability

The datasets generated and/or analyzed during the current study are available in the Gene Set Cancer Analysis (GSCA) database (https://guolab.wchscu.cn/GSCA/#/; accessed on January 15th, 2024), the UALCAN tool (https://ualcan.path.uab.edu; accessed on January 15th, 2024) and The DrugBank database (https://go.drugbank.com/; accessed on February 5th, 2024).

## Abbreviations

RPs: Ribosomal proteins
HCC: Hepatocellular carcinoma
EMT: Epithelial to mesenchymal transition
MDM2: Mouse double minute 2
5’ UTR: 5’ untranslated region
SCO2: Cytochrome c oxidase 2
HIF-1α: Hypoxia-inducible factor-1α
TLR4: Toll-like receptor 4
CDK4: Cyclin-dependent kinases 4
CDK5: Cyclin-dependent kinases 5
HBV: Hepatitis B virus
HBx: Hepatitis B virus X protein
USF1: Upstream transcription factor 1
RACK1: Receptor for activated C kinase 1
CBR1: Carbonyl reductase 1
O-GlcNAc: O-linkedβ-N-acetylglucosamine
eIFs: Eukaryotic initiation factors
FGF18: Fibroblast growth factor 18
Tcf/Lef: T cell factor/lymphoid enhancer-binding factor
FASN: Fatty acid synthase
SREBP: Sterol-regulatory element binding proteins
MMP9: Matrix metalloproteinase 9
EOC: Epithelial ovarian cancer
TIMP‑1: Tissue inhibitors of metalloproteinase-1
ROS: Reactive oxygen species
DUB: Deubiquitinating enzyme
US: Ultrasound monitoring
AFP: Alpha-fetoprotein
ssGSEA: Single sample gene set enrichment analysis
CRPs: Cytoplasmic ribosomal proteins
MRPs: Mitochondrial ribosomal proteins
mt-rRNA: Mitochondrial rRNA
TOM: Outer membrane transposase
TIM: Inner membrane transposase
DEGs: Differentially expressed genes
OXPHOS: Oxidative phosphorylation
YY1: Yin Yang 1
PD-1: Programmed cell death 1
TPT: (S)-9-dimethylaminomethyl-10-hydroxycamtothecin hydrochloride
DAMPs: Damage-associated molecular patterns
AMSCs: Adipose tissue-derived mesenchymal stem cells
